# AIDS knowledge and sexual activity among Flemish secondary school students: a multilevel analysis of the effects of type of education

**DOI:** 10.1186/1471-2458-10-30

**Published:** 2010-01-21

**Authors:** Ronan Van Rossem, Hans Berten, Charlotte Van Tuyckom

**Affiliations:** 1 Department of Sociology, Ghent University, Korte Meer 5, Ghent, Belgium

## Abstract

**Background:**

The behavior of adolescents puts them at an increased risk for HIV and other STIs, and their knowledge about HIV/AIDS is often inadequate. An understanding of how AIDS knowledge and sexual activity co-vary among Flemish secondary school students and of how education type, specifically, affects these students is limited. This study addresses the question of whether the effects of education type on HIV/AIDS knowledge and sexual activity are independent of the socio-demographic characteristics of the students.

**Methods:**

Data from the Flemish Educational Assessment survey, which collected data from a large representative sample of third- and fifth-grade high school students (*N *= 11,872), were used. Data were analyzed using multilevel logistic and Poisson regression techniques.

**Results:**

There is an indication that type of education affects both an adolescent's sexual activity and his/her AIDS knowledge; these effects prove robust for differences in socio-economic backgrounds. Students in lower status education types are more likely to be sexually active and to have poorer AIDS knowledge. The relationship between AIDS knowledge and sexual activity is, however, more complex. Although students in education types with poorer AIDS knowledge are more sexually active, within each of these groups the sexually active have better AIDS knowledge than the non-sexually active. There is also evidence of active information seeking by sexually active students, which leads to improved AIDS knowledge.

**Conclusion:**

These findings are consistent with the literature on the role of the educational system in the reproduction of social inequalities. Students from lower status education types are at increased sexual risk compared to those from higher status types. There is also evidence of active information seeking by sexually active students, which leads to improved AIDS knowledge.

## Background

In much of Europe, the big AIDS scare is over. People see AIDS as just another chronic disease and no longer see it as a deadly threat associated with "risky" sexual behavior. As the sense of threat diminishes, so does the salience of HIV/AIDS knowledge. A recent report [1] complained about the poor state of HIV/AIDS knowledge among the Belgian population and among the younger population (15-24 years) in particular. Although the risk for HIV/AIDS in the general Belgian population is relatively low and the epidemic remains largely confined to specific subpopulations, such as the Sub-Saharan African and the homosexual communities, the HIV incidence rate has been steadily climbing since the late 1990s [2]. The incidence of other STIs, such as syphilis, PID, and Chlamydia, has also risen over the last few years [2]. In more than half of the registered cases of syphilis, the patients also tested HIV positive. Many of the new AIDS cases are in their twenties, suggesting that they were infected during their late teens or early twenties.

Adolescents and young adults are at an elevated risk for STIs (including HIV) because of their sexual behavior. They tend to practice serial monogamy and are at best inconsistent condom users [3-8]. This paper examines how AIDS knowledge and sexual activity co-vary among Flemish secondary school students, and how the type of education affects this. Schools form important developmental contexts for adolescents. The education type captures important differences among schools regarding their socio-demographic composition (socio-economic and ethnic status) as well as regarding differences in educational cultures and practices. The Flemish secondary education system is highly tracked and consists of four types of education, ranging from general (ASO), artistic (KSO), and technical (TSO) to vocational (BSO) secondary education. ASO prepares students for higher education, while at the other end BSO prepares students to enter the job market directly. Students from the different types have little contact with each other. Lower status education types (TSO, BSO) tend to recruit disproportionally from the lower social strata and from minority groups, while ASO recruits more from among the middle and higher classes and among the majority population. While education in the latter is mainly formal and academic in nature, in BSO it tends to be more practice oriented and of a considerably lower level. The argument developed in this paper is that schools have effects on both AIDS knowledge and sexual behavior that are independent of these of the background characteristics of the students.

Several studies have shown a relationship between educational attainment and HIV/AIDS knowledge [3,9]. Persons with higher levels of education tend to have better HIV/AIDS knowledge. Furthermore, educational performance [10] and educational attainment [11] have also been linked with sexual risk behaviors. Little research has been done on the effects of school or educational type, but students in lower status education types (e.g., vocational education) have poorer HIV/AIDS knowledge [7,12] and display more sexual risk behaviors [4,8,10]. Other status characteristics, such as social class and ethnic group, have likewise been found to be related to differences in HIV/AIDS knowledge [8] and sexual risk behaviors [3,11,13-17]. In all these cases,	lower status groups tend to have poorer knowledge and display more risk behaviors. At the same time students from lower social classes and minority groups tend to concentrate in the lower status types of education, i.e., technical and, in particular, vocational education [14,18]. The central issue in this paper is whether the education type has an effect on a student's HIV/AIDS knowledge and sexual behavior, independent of other socio-demographic characteristics, i.e., whether differences between students from different education types merely reflect the different backgrounds of these students or if the school environment contributes something unique. The literature on the role of the education system in the reproduction of social inequality predicts the latter [19-24].

Bourdieu's idea of the habitus refers to usually class-based internalized dispositions for behavior [19]. Sexual mores and scripts can be considered part of the habitus. According to Bourdieu and Passeron [20], schools have both a selection and a socialization function: only students that possess or acquire the "right" (i.e., middle-class) cultural capital succeed, while other students flounder at lower levels of education. However, schools are not merely selection mechanisms; they also socialize students--formally and informally, intentionally and unintentionally--into particular cultures, which become part of the habitus of the students. Where the higher status types of education socialize towards higher class and middle-class cultures, the lower status types tend to socialize towards lower class cultures. Willis [21,22] takes the position that this is a more active process, in which lower class students rebel against the dominant middle-class culture of the education system and develop their own working-class culture in opposition to this dominant culture [23].

Pelleriaux [24] found similar results in the Flemish educational system: the different education types fostered distinct cultures, and the lower status types (TSO and BSO) evolved a culture of "demotion." Such a culture is characterized by feelings of being held back, a perception of future perspectives that are bleak and a general feeling that one cannot escape one's social stratum of origin, and is at odds with the dominant middle-class culture of the educational system. Important here is that the differential social backgrounds of students in the different types of education cannot explain this culture of demotion, but that it is generated and amplified within the schools and among students. The social segregation of the students in the various types of education reinforces the differences between the various types of education. Most Flemish schools offer only a single or a small number of education types, and even within schools offering multiple types, there is little interaction between students of different types, as they do not have classes together.

The Flemish secondary education system is characterized by a "waterfall" mechanism in which poorly performing students are steered towards ever "easier," and thus lower status, programs and education types. BSO students are therefore often labeled as educational failures [24,25], which affects the overall climate in vocational programs: there is little to no interest in academics or school, strained relations with teachers and administration, poor study motivation, boredom, etc. The cultures that emerge under these circumstances emphasize masculinity as a crucial element of identity, self-esteem, social status, and respect [22,26-28]. The traditional sex roles and sexual scripts [29-32] that emerge in this context stress male sexual prowess, and men feel they need to demonstrate their masculinity through conquest and sexual risk-taking. Having girlfriends or boyfriends and having sexual relations is an important status symbol for lower class students [29,31-33]. While lower class youths are known to engage in sexual relations at an earlier age and to display more sexual risk behavior [3,11,13-17], the cultures emerging within the different types of education further amplify the differences between lower class students and middle and higher class students. Consequently, one can expect students from lower status types of education, irrespective of their class or ethnic group of origin, to start their sexual career earlier than those from higher status types, to have more partners, and to practice more unsafe sex.

However, poorer AIDS-knowledge in the lower status education types not only reflects the differences in school and youth cultures, but also differences in instruction in the different types. Students in lower status education types, and in vocational education in particular, may be exposed to substantially less and poorer quality sexual education than their colleagues in general education. Where the latter receive fairly formal and academic sexual education in their biology and/or social studies classes, such approach will be less successful for the former. Not only will there be, given the cultural backgrounds of the students, greater resistance against such topics, but the school culture, the learning styles of the students and their school fatigue makes instruction much less efficient. One therefore can expect that these differences in sexual education contribute to poorer reproductive health knowledge (including AIDS knowledge) in the lower status education types.

## Methods

The data used here are from the Flemish Educational Assessment survey, in which a representative sample (N = 11.872) of students in the third and fifth grades of secondary school (equivalent to 9th and 11th grades in the American system) were interviewed in 85 randomly selected schools in Flanders during the 2004-2005 school year. The data collection was approved by the Ethics Committee of the Faculty of Political and Social Sciences of Ghent University.

We used a subsample of 9,400 students (79.1%), with valid responses on all variables, for the multivariate analyses. The variables responsible for most missing observations were sexual activity (13.8% missing), HIV/AIDS knowledge (8.8%), and family socio-economic status (6.2%).

The measure we used for HIV/AIDS knowledge was based on Zimet's [34] Adolescent AIDS Knowledge Scale. It consists of 22 items to which respondents replied using a 5-point scale (1 = statement is certainly incorrect, 5 = statement is certainly correct). A factor analysis distinguished five dimensions, accounting for 48.8% of the variance (results not shown): myths about HIV/AIDS, transmission, risk behaviors, blood transfusions, and severity. The AIDS knowledge scales were constructed by counting the number of correct answers (total or for a dimension). Because this scale represents multiple content areas, we used the split-half method to measure internal consistency, resulting in a coefficient of .76. Sexual activity was operationalized as sexually active (already had penetrative sexual intercourse) versus not (yet) sexually active.

Education type consists of four categories: general (ASO), artistic (KSO), technical (TSO), and vocational secondary education (BSO). Control variables included gender, minority status (majority vs. minority), socio-economic status (SES), study motivation, and grade. We defined minority students as students of other than West European ancestry (North African, Southern and Eastern European, etc.). A gender by minority status interaction term was included as most of the minority students were from cultures that have norms that are considerably more restrictive regarding sexuality for girls than for boys [35]. Three SES categories were distinguished based on the employment and education level of the parents: low, medium, and high. Grade is included because we can expect fifth-grade students not only to be more knowledgeable about HIV/AIDS but also more sexually experienced than third graders. Study motivation [36] was included, as earlier studies showed it to be an important determinant of academic success [37,38]. Students who were more motivated were expected to delay becoming sexually active.

## Results

### Education type and student characteristics

The results in Table 1 confirm that students are tracked into different education types according to their background characteristics. It demonstrates a clear association between education type and student SES. While 46% of the ASO students were from high-SES families, this dropped to 8% for BSO students. In contrast, the number of students from low-SES backgrounds increased from 10% for ASO to almost 50% for BSO. For minority students, we observed a similar trend: while they made up only 6% of the student body in ASO, in BSO they constituted over a quarter of the students. Girls tended to be underrepresented in TSO and overrepresented in KSO. Contrary to expectations, the study motivation varied only slightly over the different education types, with the highest mean levels found in KSO and BSO, and the lowest in TSO.

**Table 1:** Outcome and control variables by type of education

	Type of education					
			
(*s*)	ASO	KSO	TSO	BSO	Total	Explained variance
Sexually active	0.19	0.47	0.45	0.57	0.35	10.9%***
	(0.40)	(0.50)	(0.50)	(0.49)	(0.48)	
HIV/AIDS knowledge						
Myths	5.48	5.38	5.02	4.32	5.11	4.8%***
	(1.75)	(1.70)	(2.03)	(2.34)	(2.01)	
Transmission	5.33	5.30	5.09	4.61	5.12	3.7%***
	(1.11)	(1.19)	(1.46)	(1.86)	(1.42)	
Risk behaviors	3.80	3.75	3.57	3.05	3.58	3.9%***
	(1.22)	(1.33)	(1.46)	(1.73)	(1.44)	
Blood transfusions	0.69	0.64	0.54	0.39	0.58	2.4%***
	(0.77)	(0.76)	(0.72)	(0.65)	(0.74)	
Severity	0.99	1.08	0.93	0.91	0.96	0.3%***
	(0.74)	(0.72)	(0.75)	(0.78)	(0.75)	
Total	16.29	16.17	15.12	13.26	15.34	6.4%***
	(3.75)	(3.90)	(4.63)	(5.47)	(4.55)	
Study motivation	17.96	18.69	17.48	18.13	17.88	0.4%***
	(3.97)	(4.25)	(4.29)	(4.86)	(4.28)	

Categorical variables						*p*(*χ*^2^)
SES						***
Lower	10.3%	12.1%	23.3%	49.6%	21.9%	
Middle	43.5%	49.8%	56.0%	42.0%	47.0%	
Higher	46.2%	38.1%	20.8%	8.3%	31.1%	
Gender						***
Male	46.2%	38.9%	54.6%	46.7%	48.5%	
Female	53.8%	61.1%	45.4%	53.3%	51.5%	
Minority status						***
Majority	94.5%	96.0%	90.1%	74.5%	88.8%	
Minority	5.5%	4.0%	9.9%	25.5%	11.2%	

Significance: * *p *< 0.050; ** *p *< 0.010; *** *p *< 0.001

### AIDS knowledge

**Figure 1:**
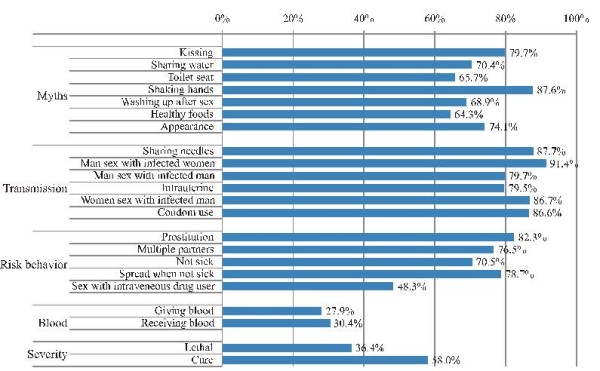
**Percentage correct answers on the AIDS knowledge items**.

On average, the respondents answered about two thirds of the HIV/AIDS knowledge items correctly. Only 1.2% of the respondents scored all items correctly, while 13% had less than half the items correct, and 1.9% did not have a single correct answer. Although a substantial minority had poor HIV/AIDS knowledge, these students showed a decent knowledge of HIV/AIDS overall. The results in Figure 1, however, show that the quality of knowledge varies substantially according to the topic. The respondents scored substantially better on items regarding the modes of transmission (85.2% correct), myths (73.0%), and risk behaviors (71.6%) than on those regarding the severity of AIDS (48.0%) and blood transfusions (29.0%). Most respondents underestimated the severity and lethality of AIDS. There were also widespread misgivings about the risks associated with blood transfusions, both donating and receiving. Most respondents considered these risky activities although the Belgian blood supply is currently quite safe.

HIV/AIDS knowledge clearly differs by education type (Table 2). ASO and KSO students scored significantly better (*p *< 0.050) on overall AIDS knowledge and on the myths, transmission, risk behaviors, and blood transfusions dimensions than BSO students, while TSO students scored in between (*p *< 0.050). The differences among the education types were less pronounced with regard only to the perceived severity of AIDS, but there as well ASO and KSO students scored significantly higher than TSO and BSO students did. We also observed that AIDS knowledge improved with age. Fifth-grade students scored significantly (*p *< 0.001) higher on both the overall HIV/AIDS knowledge scale and on all five subscales than third-grade students did. Girls on average had better HIV/AIDS knowledge than boys did. On the overall scales and on four of the subscales (myths, transmission, and risk behaviors [*p *< 0.001], and severity [*p *= 0.002]) girls scored significantly higher than boys did. They only scored worse that boys (*p *< 0.001) regarding the dangers of blood transfusions. Minority students had significantly poorer HIV/AIDS knowledge than majority students did. For the overall AIDS knowledge scale as well as for all subscales except severity, minority students scored significantly (*p *< 0.001) lower than majority students. No significant gender-minority status interaction effects were observed. Family SES was also related to AIDS knowledge. High-SES students on average scored better on all AIDS knowledge variables than middle-SES students, who in turn scored better than low-SES students did. All pairwise differences were significant at *p *< 0.001, except for the one between high- and middle-SES students on the severity subscale (*p *= 0.025). Study motivation was weakly but significantly correlated with the overall AIDS knowledge scale (*r *= -0.03, *p *= 0.004) and two of the subscales: myths (*r *= -0.05, *p *< 0.001) and blood transfusions (*r *= -0.03, *p *= 0.001).

**Table 2:** AIDS knowledge by education type and sexual activity of respondent

			Education type				
				
			ASO	KSO	TSO	BSO	Total
3^rd ^grade	Not sexually active	M	15.3	15.3	13.5	11.3	14.4
		SD	(3.9)	(4.1)	(4.8)	(5.4)	(4.5)
		N	*2474*	*91*	*866*	*466*	*3897*
		P	*(90.7%)*	*(71.1%)*	*(72.7%)*	*(56.5%)*	*(80.0%)*
	
	Sexually active	M	15.8	15.3	14.4	12.6	14.1
3^rd ^grade		SD	(3.9)	(4.6)	(4.5)	(5.4)	(4.9)
		N	*255*	*37*	*326*	*359*	*977*
		P	*(9.3%)*	*(28.9%)*	*(27.3%)*	*(43.5%)*	*(20.0%)*
	
	Total	M	15.4	15.3	13.8	11.8	14.4
		SD	(3.9)	(4.2)	(4.8)	(5.4)	(4.6)
		N	*2729*	*128*	*1192*	*825*	*4874*

5^th ^grade	Not sexually active	M	17.4	16.5	15.7	13.6	16.4
		SD	(3.1)	(3.4)	(4.4)	(5.4)	(4.1)
		N	*1489*	*55*	*743*	*364*	*2651*
		P	*(68.5%)*	*(37.7%)*	*(44.1%)*	*(32.5%)*	*(51.7%)*
	
	Sexually active	M	17.7	17.1	16.4	14.8	16.3
5^th ^grade		SD	(3.1)	(3.3)	(4.0)	(5.1)	(4.3)
		N	*686*	*91*	*941*	*757*	*2475*
		P	*(31.5%)*	*(62.3%)*	*(55.9%)*	*(67.5%)*	*(48.3%)*
	
	Total	M	17.5	16.9	16.1	14.4	16.4
		SD	(3.1)	(3.3)	(4.2)	(5.2)	(4.2)
		N	*2175*	*146*	*1684*	*1121*	*5126*

M: mean; SD: standard deviation; N: cell size; P: proportion of grade

Proportion of variance explained in AIDS knowledge: 3rd grade: 8.8%, *p *= 0.000; 5th grade: 8.9%, *p *= 0.000

### Sexual activity

Table 1 shows that about 35% of the sample were already sexually active. ASO students were significantly less likely to be sexually active than the others, while BSO students were most likely to be sexually active. Of course, the proportion of sexually active students increased substantially from the third grade to the fifth grade (see Table 2). While only one fifth of third-grade students reported having had sex, this rose to almost half in the fifth grade (20.0% vs. 51.7%, χ^2^[1] = 895.1, *p *= 0.000). There was a negative relation between the students' family SES and their sexual activity. Lower SES students were not only sexually active earlier but were also more often sexually active than higher SES students (χ^2^[2] = 118.2, *p *= 0.000).

Overall, over the two grades there were a few differences regarding sexual activity based on gender or minority status (results not shown). Boys were somewhat more likely to start their sexual career early than girls were. For boys in the third grade, 22.2% reported being sexually active compared to only 18.8% of girls (*p *= 0.003). By the fifth grade, this difference had disappeared. However, the gender differences regarding sexual activity among minority status respondents were even more interesting. While there were no meaningful gender differences regarding sexual activity among majority students, clear gender differences emerged among minority students. While in the third grade 49.5% of the minority boys reported already being sexually active, only 14.8% of the girls reported the same (*p *= 0.000). In the fifth grade the proportion of sexually active minority boys versus girls had risen to 59.7% and 25.5%, respectively, (*p *= 0.000). These results clearly indicate the different standards for minority (mainly Muslim) boys versus girls. We also observed a small but significant negative correlation between study motivation and sexual activity (*r *= -0.08, *p *= 0.000).

### Association AIDS knowledge, sexual activity

Overall, sexually active respondents were observed to have better HIV/AIDS knowledge than non-sexually active ones. Their mean scores on the total AIDS knowledge scale were, respectively, 15.70 (*s *= 4.58) and 15.23 (*s *= 4.46) (*p*_diff _= 0.000). Except for contagion, we found similar results for the various subscales (results not shown). This finding, however, is largely an artifact, due to the mix of third- and fifth-grade students, the latter not only being more sexually active but also having better knowledge about AIDS. When we split the sample by grade (Table 2), we found that the differences between sexually active and non-sexually active students were no longer significant, except for the transmission subscale among third graders. However, as Table 2 shows, such a conclusion is also misleading. These results show that there is a clear relationship between AIDS knowledge and sexual activity when the sample is further broken down by education type. Within each education type, except for KSO, sexually active students had significantly (all *p *= 0.000) better AIDS knowledge (both total and various dimensions) than non-sexually active ones. However, as the proportion of sexually active students was higher in the lower status education types, and in BSO in particular, and the latter also tended to have poorer AIDS knowledge, an overall negative association between sexual activity and AIDS knowledge emerged. This negative association is compensated for, only to an extent, by better knowledge about AIDs by sexually active students within each of the education types. The AIDS knowledge of sexually active BSO students, however, was still significantly lower (at *p *< 0.000) than that of the non-sexually active students in the other education types. The relationship between AIDS knowledge and sexual activity, therefore, is quite complex. On the one hand, there are groups with poorer AIDS knowledge that are more sexually active, but within each of these groups the sexually active have better AIDS knowledge than the others.

### Multivariate analysis

Because cluster sampling methods were used to collect the data and the dependent variables were either dichotomous or counts, multilevel logistic and Poisson regression techniques were used for the multivariate analysis. The findings in Table 3 and Table 4 confirm that the effects of education type are robust for the effects of the control variables. With ASO as reference group, students in all other education types were significantly more likely to engage in sexual activity. BSO students, in particular, were--even after controlling for gender, majority status, socio-economic background, school motivation, and grade--significantly more likely than ASO students to engage in sexual activity (OR_BSO/ASO _= 5.48). KSO and TSO students also remained more likely to be sexually active, but for them the effect was more moderate (OR_KSO/ASO _= 2.61; OR_TSO/ASO _= 3.03).

**Table 3:** Multilevel regression results for sexual activity and total AIDS knowledge

b (se)	Sexually active^1^		AIDS knowledge (total)^2^	
Constant	-1.443*** (0.086)	-3.854*** (0.203)	2.769*** (0.015)	2.503*** (0.027)
Education type (ref: General)				
Artistic	0.860*** (0.198)	0.959*** (0.232)	-0.104** (0.034)	-0.089** (0.034)
Technical	1.176*** (0.110)	1.110*** (0.110)	-0.061** (0.018)	-0.069*** (0.015)
Vocational	1.663*** (0.106)	1.702*** (0.105)	-0.167*** (0.028)	-0.160*** (0.025)
Grade		0.687*** (0.033)		0.070*** (0.005)
Sex: Female		0.240*** (0.057)		0.677*** (0.098)
Minority status		2.209*** (0.308)		-0.172* (0.077)
Interaction sex & minority status		-1.749*** (0.219)		0.004 (0.043)
Study motivation		-0.045*** (0.006)		-0.002* (0.001)
SES (ref: High)				
Low		-0.026 (0.089)		-0.058*** (0.011)
Medium		0.168* (0.069)		-0.029*** (0.007)

Variance components				
School level	0.238***	0.209***	0.012***	0.006***

Significance: * *p *< 0.050; ** *p *< 0.010; *** *p *<0.001

^1 ^Multilevel logistic regression estimates

^2 ^Multilevel poisson regression estimates

**Table 4:** Multilevel Poisson regression results for the various AIDS knowledge dimensions

b (se)	Myths	Transmission	Risk behaviors	Blood transfusions	Severity
Constant	1.358*** (0.032)	1.438*** (0.023)	1.050*** (0.033)	-0.808*** (0.092)	-0.307*** (0.053)
Education type (ref: General)					
Artistic	-0.075* (0.036)	-0.032 (0.018)	-0.048 (0.032)	-0.154 (0.104)	0.032 (0.071)
Technical	-0.089*** (0.015)	-0.040*** (0.009)	-0.062*** (0.016)	-0.269*** (0.039)	-0.086** (0.030)
Vocational	-0.188*** (0.024)	-0.110*** (0.021)	-0.174*** (0.031)	-0.488*** (0.061)	-0.097* (0.044)
Grade	0.088*** (0.007)	0.036*** (0.005)	0.069*** (0.006)	0.201*** (0.017)	0.084*** (0.011)
Sex: Female	0.066*** (0.009)	0.062*** (0.007)	0.035*** (0.008)	-0.117*** (0.024)	0.030 (0.019)
Minority status	-0.192* (0.084)	-0.217** (0.078)	-0.175 (0.104)	-0.051 (0.219)	-0.008 (0.139)
Interaction sex & minority status	-0.006 (0.047)	0.041 (0.041)	-0.027 (0.062)	-0.210 (0.160)	0.012 (0.084)
Study motivation	-0.004*** (0.001)	0.001 (0.001)	0.000 (0.001)	-0.007* (0.003)	-0.003 (0.002)
SES (ref: High)					
Low	-0.069*** (0.012)	-0.034** (0.010)	-0.070*** (0.016)	-0.190*** (0.046)	-0.067* (0.029)
Medium	-0.030** (0.009)	-0.020** (0.007)	-0.030** (0.009)	-0.086** (0.028)	-0.052** (0.017)
Variance components					
School level	0.005***	0.001***	0.003***	0.014***	0.007***

Significance: * *p *< 0.050; ** *p *< 0.010; *** *p *< 0.001

The AIDS knowledge of students in KSO, TSO, or BSO also remained significantly poorer than that of ASO students, even after controlling for the other variables in the model. Again, BSO students performed worst; their total AIDS knowledge was on average only 85% of that of otherwise similar ASO students. For TSO and KSO students, total AIDS knowledge was only 93% and 91%, respectively. For the various subscales, a similar pattern was seen, except that the difference between KSO and ASO students was no longer significant for most of them, due to the small number of KSO students in the sample.

That fifth-grade students were more sexually active and had better AIDS knowledge than those in the third grade does not come as a surprise. Girls were also more likely to be sexually active and to have better AIDS knowledge. Minority boys, but not girls, also had a significantly higher probability of being sexually active. Minority students also had significantly poorer AIDS knowledge than majority ones. On the subscales, this effect was significant only for the myths and transmission scales, but the effects on the other scales were in the same direction. No gender-minority status interaction effect was detected regarding AIDS knowledge.

The socio-economic status of the students, controlling for the other variables, had little impact on whether or not they were sexually active. The socio-economic background of the students, however, had a more important effect on AIDS knowledge. The lower the SES of the student, the poorer his or her AIDS knowledge was. Study motivation was found to negatively affect not only the likelihood of being sexually active, but also AIDS knowledge.

## Discussion

The type of education a Flemish adolescent attends affects not only the likelihood of being sexually active, but also his or her knowledge about AIDS. These effects prove robust when controlling for other socio-demographic characteristics of the respondents. Students in lower status education types and those in BSO in particular are more inclined to have sex early and tend to have poorer AIDS knowledge than ASO students do. However, the exact nature of the relationship between sexual activity and AIDS knowledge remains unclear as within each type of education sexually active students have better AIDS knowledge than non-sexually active ones, while the likelihood of being sexually active is highest among the groups with the poorest AIDS knowledge.

HIV/AIDS knowledge may also be seen as a proxy for general reproductive health knowledge. The observation that youths who start their sexual careers earlier have, on average, poorer reproductive health knowledge than those who delay the onset may indicate that the former group is at increased risk, not only for HIV/AIDS, but also for other STIs and for unwanted pregnancy. On a more positive note, we find that within each type of education the sexually active students have better AIDS knowledge than non-active ones. This may indicate active information seeking on the part of sexually active students or may indicate an increased receptiveness in these students to reproductive health messages, since this information becomes more personally relevant. For students who are not sexually active, knowledge about reproductive health matters may be less pertinent.

The analyses show that education type has a distinct effect on both the sexual behavior of the respondents and on their knowledge about AIDS. This effect of education type is not reducible to the differences in the backgrounds of the students. The education type reinforces influences from the students' backgrounds. BSO students therefore are at a double risk, from their background, as they are recruited disproportionally from minority and low-SES families, and from their school environment. This finding is consistent with the literature on the role of the education system in the reproduction of social inequalities [22-24]. Schools (re)produce youth cultures that "prepare" students for their future roles in society. Although some of this cultural reproduction is intended and is part of the curriculum and school culture, much of it is an unintended--but recognized--consequence of the interactions among students and between students and staff. In the lower status education types and in BSO in particular these emerging cultures stress masculinity as an informal status dimension, which is reflected in the sexual norms and scripts. These youth cultures incorporate elements of the overall culture as well as of the cultures of the dominant groups within the schools. As there is social segregation by education type, the cultures vary substantially over the various education types. While students in ASO mainly come from the majority group--middle to higher classes--the BSO students are mainly minority and lower class. The youth cultures that emerge in these different types of education reflect this.

A limitation of this study is that it is based on self-report and that a social desirability bias may affect the results for sexual activity. For instance, Muslim communities strongly curtail sexuality, especially for females, which may have lead to underreporting or non-response on this variable. Another limitation is the use of cross-sectional data. This makes it difficult to establish causality, especially with regard to the relation between AIDS knowledge and sexual activity, but also regarding whether the relationship with education type is due to self-selection or socialization. For instance, we have no information on the students' reproductive health knowledge in general and AIDS knowledge in particular prior to entering high school. One can expect this knowledge to be related to overall academic performance in elementary school and thus also to the tracking in the different education types. Both the knowledge and the tracking are also related to the background characteristics of the children, mainly SES and ethnicity. For the backgrounds of the students we controlled in the multivariate analysis, and to the extent that differences in knowledge levels and academic performance contribute to the tracking of students, they form constituent parts of the specific cultures that develop in the various education types.

## Conclusion

This study emphasizes that the type of education affects both the onset of sexual activity and AIDS knowledge, and that these effects prove robust for differences in socio-economic backgrounds of the students. Furthermore, the relationship between AIDS knowledge and sexual activity is quite complex and sexually active students may engage in active information seeking.

Our results have implications for reproductive health education and campaigns. Flemish schools do not provide comprehensive reproductive health education but cover these issues in a range of courses. Our results suggest that schools need to develop a differentiated approach according to type of education and that they should give more attention to reproductive health education in lower status education types. Education and learning styles differ substantially among the education types. While ASO programs may present reproductive health knowledge in a more academic fashion (e.g., lectures), reproductive health classes in BSO need to be oriented more towards the everyday practices of their students. There is also evidence that the way reproductive health information is provided in schools is not popular, especially among minority teens with a Muslim background, which are disproportionally overrepresented in these lower educational tracks [35]. Further, ASO and BSO students differ substantially with regard not only to the amount of information they receive, but also to their need for such information. This points to the need for the development of sexual education curricula adapted to the needs of TSO and, especially, BSO students, that take into account the specific cultures of these schools. Additionally, such programs should foster the information seeking and processing skills of the students. This would allow them, when they become sexually active, to be more effective in looking for relevant and accurate information regarding sexuality and reproductive health issues.

## Competing interests

The authors declare that they have no competing interests.

## Authors' contributions

RVR collected the data, conducted all statistical analyses, wrote, and coordinated the paper. HB provided theoretical additions and other comments to the manuscript, and CVT provided statistical support. All authors commented on drafts, have read the revised manuscript, and have approved the final version.

## Pre-publication history

The pre-publication history for this paper can be accessed here:

http://www.biomedcentral.com/1471-2458/10/30/prepub
